# Erythrocyte selenium concentration is a predictor of mortality in patients with septic shock

**DOI:** 10.1186/cc12663

**Published:** 2013-06-19

**Authors:** NA Costa, BLB Pereira, AL Gut, PS Azevedo, LAM Zornoff, SAR Paiva, MF Minicucci

**Affiliations:** 1 Botucatu Medical School, UNESP, Rubião Júnior, Botucatu, SP, Brazil

## Introduction

Severe sepsis and septic shock are major healthcare problems, affecting millions of people around the world each year, and increasing in incidence [1]. During sepsis there is increased oxidative stress, with reduced body stores of selenium (Se) and lower activity of glutathione peroxidase (GPx1), with patient outcomes of multiple organ failure and death [2]. The objective of this study was to determine the influence of Se concentration in plasma, erythrocytes and erythrocyte GPx1 activity on the length of hospital stay, length of ICU stay and ICU mortality in patients with septic shock.

## Methods

This prospective study included all patients with septic shock on admission or during ICU stay, over the age of 18, admitted to one of the three ICUs of the Botucatu Medical School, from January to August 2012. Demographic information, clinical evaluation and blood samples were taken within the first 72 hours of the patient's admission or within 72 hours after septic shock diagnosis for laboratory analysis, GPx activity, plasma and erythrocyte Se concentration. Categorical variables were analyzed by chi-squared or Fisher exact test. Continuous variables were analyzed by Student's *t *test. For length of ICU or hospital stay, prediction multiple linear regression was used. For mortality prediction, multiple logistic regression was performed. The level of significance was set at 5%.

## Results

We evaluated 110 patients with a mean age of 58 ± 16 years and 63% were male. The median length of ICU stay and hospital stay was 9 (5 to 15) and 18 (11 to 34) days, respectively, and the ICU mortality rate was 55%. Patients had an average plasma and erythrocyte Se concentration of 23.37 ± 8.99 and 32.83 ± 11.89 g/l, respectively, and the median GPx1 activity was 30.56 (23.98 to 38.41) U/g Hb. All patients had Se deficiency; however, only 25% had reduced GPx activity. There was no association of plasma and erythrocyte Se concentration and GPx activity with the length of hospital and ICU stay. Higher values of APACHE II, albumin and creatinine were associated with higher erythrocyte Se concentration. Regarding mortality, it was associated with higher lactate, urea, APACHE II and SOFA scores and lower values of albumin and length of hospital and ICU stay. In multiple logistic regression analysis adjusted for age, gender, and APACHE II, the erythrocyte Se concentration was a predictor of mortality in patients with septic shock (OR: 0.922; 95% CI: 0.880 to 0.967; *P *<0.001).

## Conclusion

The erythrocyte Se concentration is a predictor of mortality in patients with septic shock.

## References

[B1] Linde-Zwirble WT Angus DC Severe sepsis epidemiology: sampling, selection, and society Crit Care 2004 17 222 226 10.1186/cc2917 15312201 PMC522859

[B2] Heyland DK Dhaliwal R Suchner U Berger M Antioxidant nutrients: a systematic review of vitamins and trace elements in the critically ill patient Int Care Med 2005 17 327 337 10.1007/s00134-004-2522-z 15605227

